# The Pandemic Year 2020: World Map of Coronavirus Research

**DOI:** 10.2196/30692

**Published:** 2021-09-08

**Authors:** Doris Klingelhöfer, Markus Braun, Dörthe Brüggmann, David A Groneberg

**Affiliations:** 1 Institute of Occupational, Social and Environmental Medicine Goethe University Frankfurt Germany

**Keywords:** COVID-19, SARS-CoV-2, incidence, research funding, socioeconomic factors, bibliometrics, bibliometric analysis, global health, public health, health database, online research, research database

## Abstract

**Background:**

SARS-CoV-2 is one of the most threatening pandemics in human history. As of the date of this analysis, it had claimed about 2 million lives worldwide, and the number is rising sharply. Governments, societies, and scientists are equally challenged under this burden.

**Objective:**

This study aimed to map global coronavirus research in 2020 according to various influencing factors to highlight incentives or necessities for further research.

**Methods:**

The application of established and advanced bibliometric methods combined with the visualization technique of density-equalizing mapping provided a global picture of incentives and efforts on coronavirus research in 2020. Countries’ funding patterns and their epidemiological and socioeconomic characteristics as well as their publication performance data were included.

**Results:**

Research output exploded in 2020 with momentum, including citation and networking parameters. China and the United States were the countries with the highest publication performance. Globally, however, publication output correlated significantly with COVID-19 cases. Research funding has also increased immensely.

**Conclusions:**

Nonetheless, the abrupt decline in publication efforts following previous coronavirus epidemics should demonstrate to global researchers that they should not lose interest even after containment, as the next epidemiological challenge is certain to come. Validated reporting worldwide and the inclusion of low-income countries are additionally important for a successful future research strategy.

## Introduction

In December 2019, a new coronavirus (CoV) variant infected some patients in China. It was transmitted at a seafood and wet animal wholesale market in Wuhan city, Hubei Province. This novel zoonotic coronavirus was named SARS-CoV-2 because it also causes severe acute respiratory syndrome (SARS) [1]. Previously detected coronaviruses such as SARS-CoV and Middle East Respiratory Syndrome (MERS)-CoV led to temporary pandemics and consequently to serious public health challenges, which, however, came to an end with the containment of the diseases and their spread. The first cases of the infectious disease caused by SARS-CoV-2, designated COVID-19, indicated the beginning of an outbreak that would become a still ongoing global pandemic on a scale not seen since the Spanish flu in 1918, which killed up to 50 million people [2].

As of this analysis (January 12, 2021), more than 93 million COVID-19 cases have been confirmed, with the number continuing to rise rapidly. Over 2 million people have died in association with SARS-CoV-2 infection as of that date [3]. The enormous impact is catastrophic and affects all areas of public, political, economic, and private life. For sure, it will for a long time. The associated demands and restrictions on citizens and the social systems of all nations are undeniable.

Indeed, it was expected that the number of publications on CoV would increase sharply in 2020, but to what extent and with what participation were not clear [4]. There already have been some studies on the general output of COVID-19–related publications [5-7]. In addition, however, it is necessary to identify and evaluate the general and national research efforts according to additional influences such as epidemiological and funding characteristics to enable successful and determined planning, funding, and implementation of science-based research in the future that reaches all necessary areas through balanced multidisciplinary research.

To achieve this objective, this study mapped the world according to various influencing factors, leading to an advanced and meaningful assessment of the research of the first COVID-19 pandemic year 2020, which will certainly not be the last.

## Methods

### Methodological Platform and Data Source

This study followed the methodological approach of the bibliometric platform New Quality and Quantity in Science (NewQIS) [8]. For the first time, NewQIS combined bibliometric analyses with density-equalizing mapping (DEMPs) [9] to depict the global publication landscape on scientific topics. DEMPs enable the rapid acquisition of large-scale data. In this process, countries are distorted according to the density-equalizing principle applied by an algorithm developed by Gastner and Newman [9]. The result is a distorted world map according to the respective evaluation parameter, with countries with high values enlarged and countries with low values reduced.

The aim was to provide solid information on research patterns in terms of trends, incentives, challenges, obstacles, and necessities for all parties involved. Socioeconomic parameters and research-specific conditions at the country level were included in the analyses to assess regional performance according to the need for valuable and balanced research that is accessible and appropriate for all parts of the world.

### Search Strategy

To capture all CoV-related articles published in 2020, the following elaborated search term was applied in the Web of Science Core Collection (WoS) search field: Title: “corona virus*” OR “coronavirus*” OR “SARS” OR “MERS” OR “covid-19” OR “severe acute respiratory syndrome” OR “middle east respiratory syndrome” AND Topic: virus* OR epidem* OR CoV OR Co-V OR patient* OR outbreak* OR “corona virus” OR “coronavirus” OR “covid-19” OR “severe acute respiratory syndrome” OR “middle east respiratory syndrome”. This string ensured the representativeness of the database generated. Then, the entries were filtered by original articles to base the evaluation on actual research on coronavirus. No language filter was applied. The year 2020 was chosen as the time frame. The date of data collection was January 12, 2021.

The metadata of the datasets collected in this way were stored and sorted according to the individual evaluation parameters and linked by assigning identification numbers to each entry. Some parameters had to be additionally corrected manually, such as matching institution names and funding sources.

### Utilized Parameters and Analyses

In addition to established bibliometric parameters such as publication performance, citation parameters, and networking, CoV-specific parameters were also analyzed in this study. These relate to epidemiological characteristics (numbers of cases associated with COVID-19 [3]), socioeconomic characteristics (gross domestic product [GDP], population size), and funding characteristics of the publishing countries. The socioeconomic and epidemiological parameters were used as absolute numbers to allow comparison with absolute publication numbers. The use of per capita values would also require the use of publication figures per capita, which would be redundant when calculating the ratio. International collaborations were defined by the participation of at least two countries of origin, as indicated in the author's affiliations. China and Taiwan were analyzed separately.

In addition, an analysis of the development of publication and citation numbers in 10 time intervals, into which the year 2020 was divided, was carried out. Furthermore, an analysis of research areas was performed employing clustering with the application VOSviewer developed by van Eck and Waltman [10]. Author keywords were clustered and displayed by nodes and connecting lines that represent the different research areas.

### Methodological Limitations

Although the methodology used provides a valid source of data, some limitations must be considered when evaluating the results. First, all analyses can only be as good as the database used. For all NewQIS studies, WoS serves as the standard data source. Despite the often-documented English bias and the limitation of a somewhat restricted dataset, according to the high indexing requirements [11], the database provides representative and qualitative results for the further analyses of this study [12]. Some entries had to be manually unified, such as funding sources, which is a nonrepeatable, standardized procedure. In addition, citation-based analyses are prone to error and cannot be considered a proxy for research quality. Nevertheless, the combination of applied ones provides a deep insight into publication performance.

## Results

### Main Research Foci of CoV Research in 2020

COVID-19 as the most frequently occurring keyword indicates that almost all coronavirus research in 2020 related to SARS-CoV-2 infection, as confirmed by a manually performed review of the articles included in the database.

**Figure 1:**
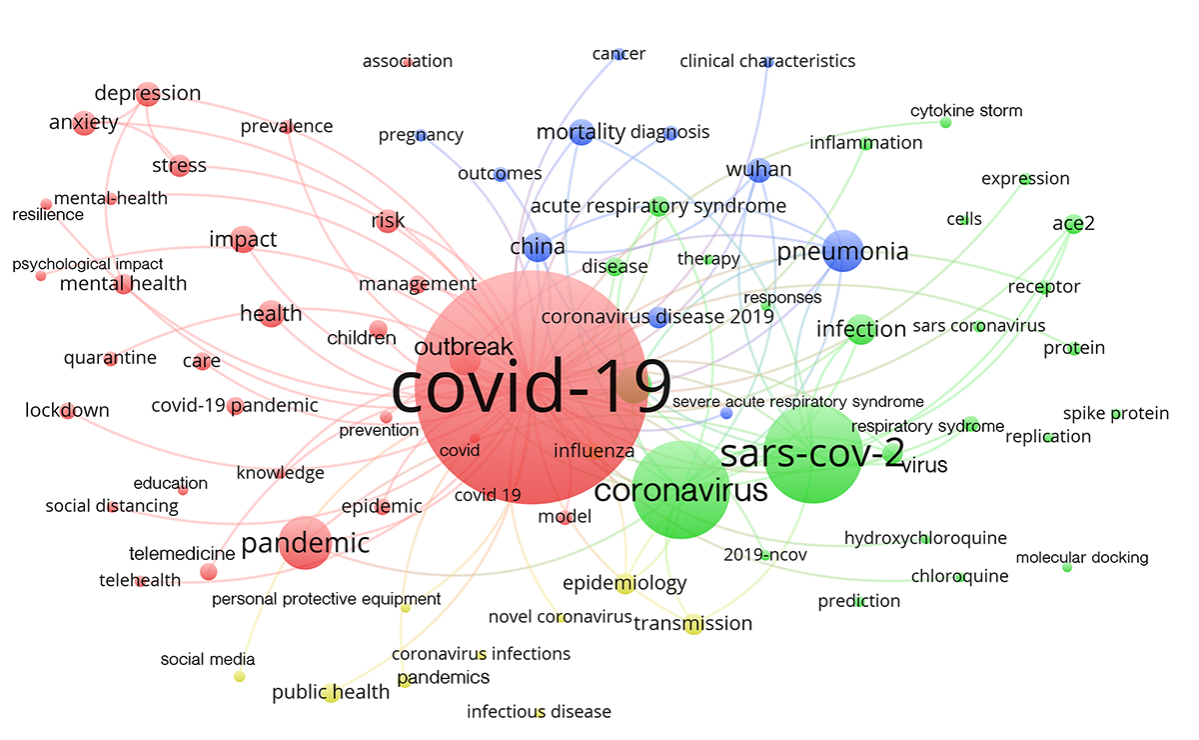
Clusters of keywords (threshold: 200 occurrences). Red: psychological health effects; blue: physical health effects and mortality; green: biochemistry and cell biology; yellow: epidemiology and public health.

In addition, analysis of the most frequently used keywords (threshold: 200 occurrences) revealed 4 thematic clusters dealing with the psychological and physical impacts of COVID-19, immunological and biochemical issues, and epidemiological and public health issues (Figure 1). The articles could mainly be assigned to the WoS categories “General and Internal Medicine” (n=8488), “Public, Environmental & Occupational Medicine” (n=2845), “Infectious Diseases” (n=1830), “Science & Technology – Other Topics” (n=1671), “Environmental Science & Ecology” (n=1397), and “Psychiatry” (n=1116).

### Evolution of CoV Research in 2020

**Figure 2:**
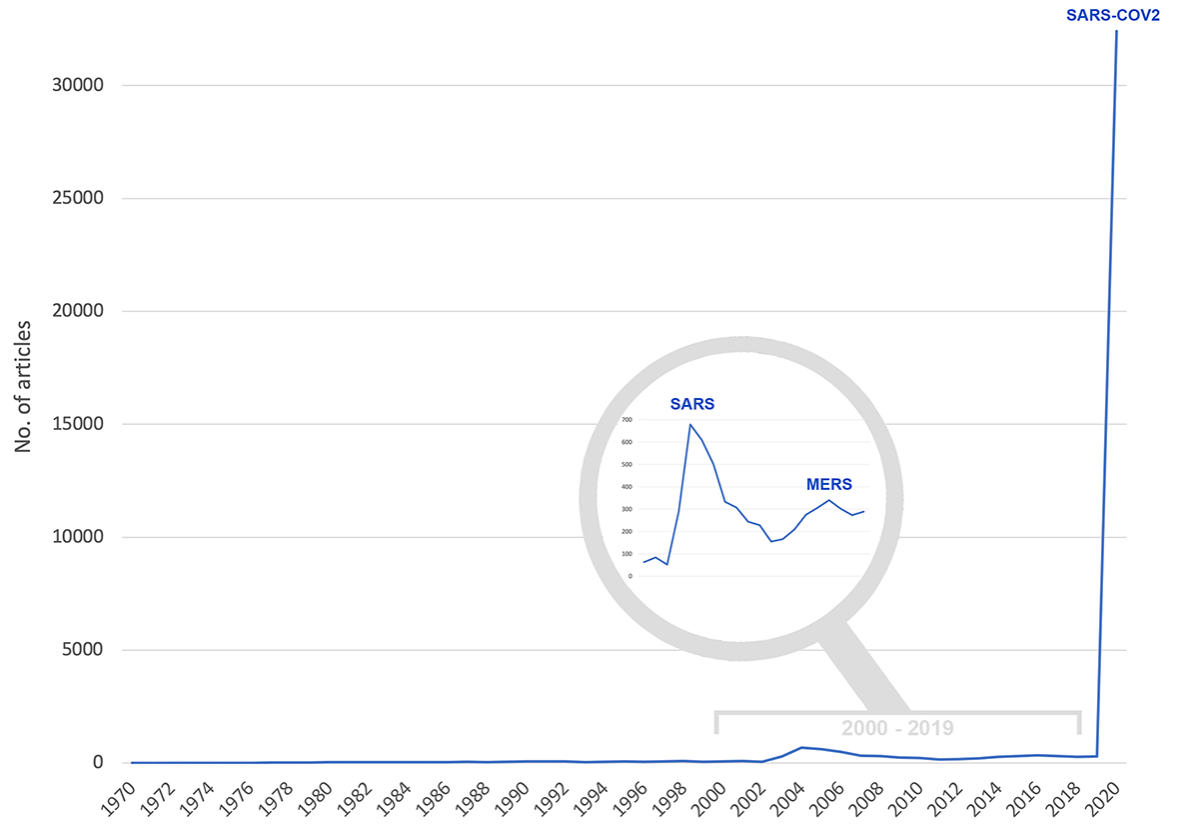
Development of the numbers of articles about coronavirus research. The comparative figures from 1970-2019 are taken from a previous study by Klingelhöfer et al [4] to show the immense increase in publication numbers in 2020. MERS: Middle East Respiratory Syndrome; SARS: Severe Acute Respiratory Syndrome.

Applying search terms yielded 67,437 publications on CoV in 2020, of which just under half were original articles (n=32,402) that comprised the analysis database. Of the other document types, 12,435 were letters, 12,245 were editorial material, 6956 were classified as early access, and 6556 were reviews, to name the most common publication types. Looking at the overall evolution of CoV research, the year 2020 was exorbitantly outstanding (Figure 2). The epidemics of SARS in 2003 and MERS in 2012 caused the number of articles to increase to 679 in 2004 and to 340 in 2016, which decreased as soon as the epidemics were contained [4]. These figures show that the number of articles on CoV in 2020 was almost 50 times higher than in the previous peak year of 2004.

Although the 2020 articles are still very young to generate a significant number of citations and thus recognition in the scientific community, which underlies a citation half-life of 7-8 years for biomedical articles, some articles already received an exceptionally high number of citations. The 10 most frequently cited articles are summarized in Table 1. They were published in high-impact journals and are predominantly from China, where SARS-CoV-2 first appeared. Two international collaborations are also represented in the ranking: 1 is an international collaboration of German, Austrian, and Russian scientists, and the other is a Chinese-Australian partnership. All received financial support through government programs from China or Germany.

**Table 1:** Most frequently cited articles on coronavirus (CoV) in 2020 (as of January 12, 2021).

Authors (location)	Number of citations	Title	Source
Huang et al [13] (China)	8978	Clinical features of patients infected with 2019 novel coronavirus in Wuhan, China	The Lancet
Guan et al [14] (China)	5824	Clinical characteristics of coronavirus disease 2019 in China	NEJM^a^
Wang et al [15] (China)	5353	Clinical characteristics of 138 hospitalized patients with 2019 novel coronavirus-infected pneumonia in Wuhan, China	JAMA^b^
Zhou et al [16] (China)	4987	Clinical course and risk factors for mortality of adult inpatients with COVID-19 in Wuhan, China: a retrospective cohort study	The Lancet
Zhu et al [17] (China)	4865	A novel coronavirus from patients with pneumonia in China, 2019	NEJM
Chen et al [18] (China)	4525	Epidemiological and clinical characteristics of 99 cases of 2019 novel coronavirus pneumonia in Wuhan, China: a descriptive study	The Lancet
Zhou et al [19] (China)	3762	A pneumonia outbreak associated with a new coronavirus of probable bat origin	Nature
Li et al [20] (China)	3101	Early transmission dynamics in Wuhan, China, of novel coronavirus-infected pneumonia	NEJM
Hoffmann et al [21] (Germany, Austria, Russia)	2717	SARS-CoV-2 cell entry depends on ACE2 and TMPRSS2 and is blocked by a clinically proven protease inhibitor	Cell
Lu et al [22] (China, Australia)	2439	Genomic characterisation and epidemiology of 2019 novel coronavirus: implications for virus origins and receptor binding	The Lancet

^a^NEJM: New England Journal of Medicine.

^b^JAMA: Journal of the American Medical Association.

### Highest Publishing Countries on CoV in 2020

**Figure 3:**
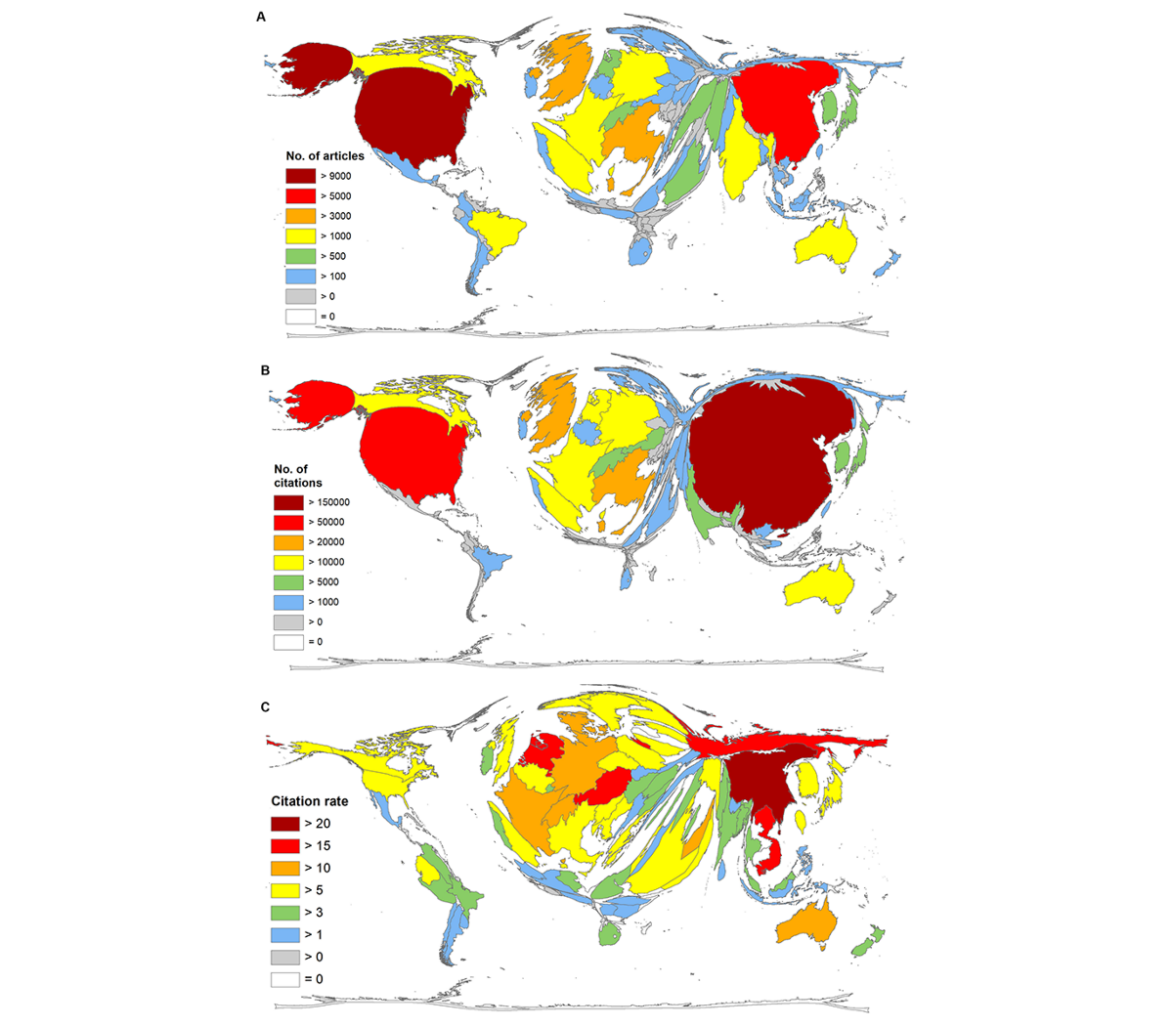
Density-equalizing maps showing the (A) number of articles, (B) number of citations, and (C) citation rate (citations per article), threshold >30 articles.

In total, 170 countries or autonomous regions participated in research on CoV in 2020 that was indexed in WoS. By far, the United States had the most publications (n=9018), followed by China (n=5053), Italy (n=3195), the United Kingdom (n=3135), and India (n=1847; Figure 3A).

In contrast, China received the most citations (n=155,522). The United States got considerably fewer citations on their CoV-related articles (n=86,003). It is followed by the United Kingdom (n=29,840), Italy (n=28,530), and Germany (n=18,695). India ranked only 12th with 6351 citations (Figure 3B).

Resulting from these numbers, China also led the ranking when looking at the citation rates of countries with at least 30 articles on CoV in 2020 (threshold) with a citation rate of 30.78. It was followed by the Netherlands (citation rate=19.56), Russia (citation rate=17.04), Austria (citation rate=16.18), and Vietnam (citation rate=16.07). In this term, the United States and the United Kingdom ranked nearly similarly, at only 22nd (citation rate=9.54) and 23rd (citation rate=9.52), and India was even only ranked at 79th (citation rate=3.44; Figure 3C).

**Figure 4:**
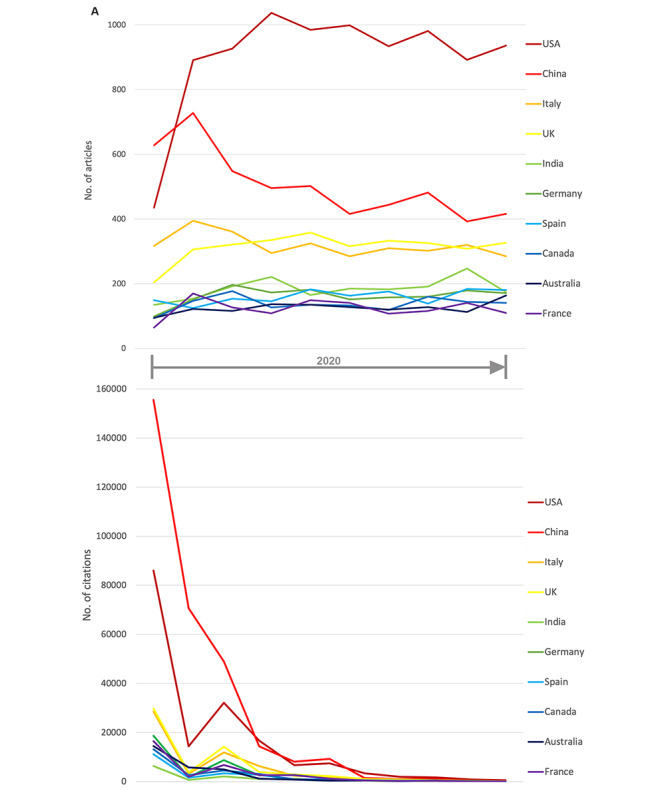
Development in 2020 of the 10 countries with the highest number of articles, including the (A) number of articles on the topic of coronavirus and (B) number of citations of the articles on the topic of coronavirus.

The dominance of US-American and Chinese researchers in terms of CoV research over the entire year 2020 could be shown by the numbers of articles and citations. Nevertheless, their share varied over the year. While Chinese articles dominated in the early phase, US-American articles gained momentum as the year progressed. The United Kingdom’s share also increased during the year, eventually overtaking the share of Italy (Figure 4A). The same holds true for the trend in the number of citations, although this depends on the minimum time the articles had to generate citations by other publications (Figure 4B).

**Figure 5:**
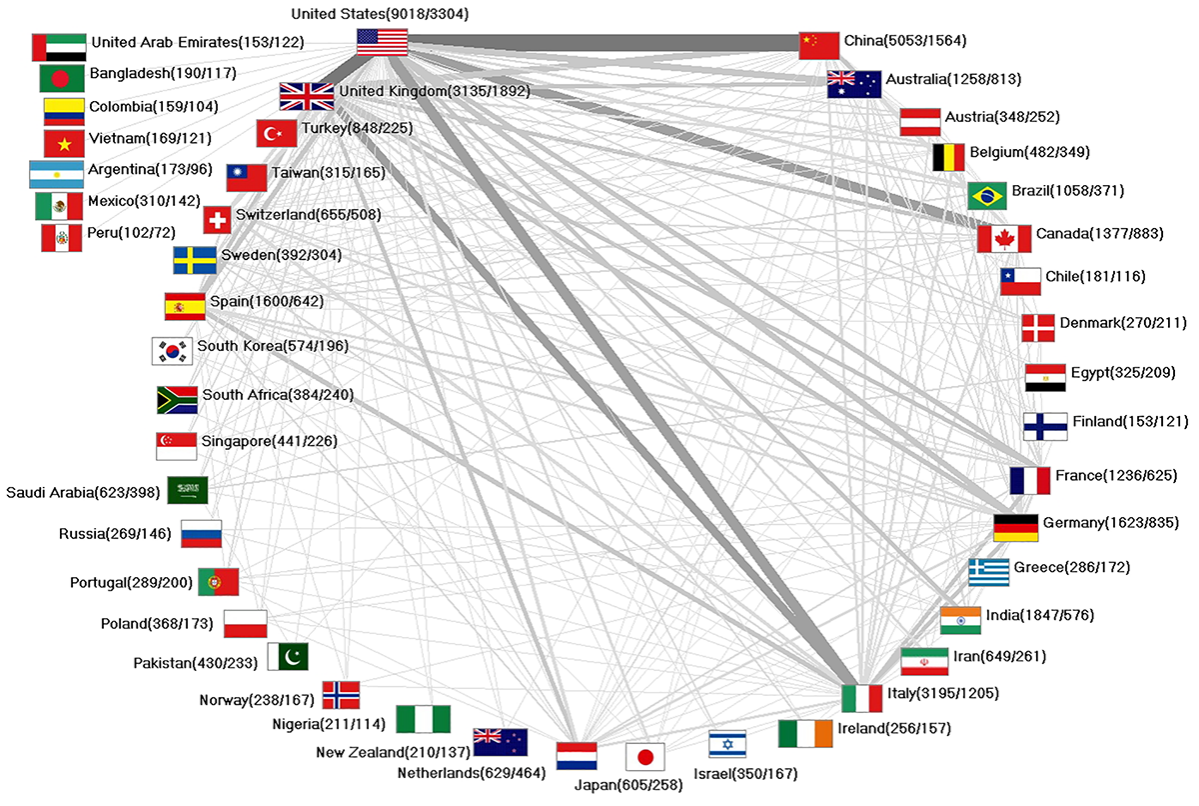
International network on coronavirus (threshold 35 collaborations).

A broad international network on CoV research has developed, with the United States and China being the main cooperation countries with 800 collaborations (Figure 5). In general, the United States acts as the main core country when considering international partnerships. In addition to the Chinese collaborations, there were 640 collaborations with the United Kingdom, 496 collaborations with Italy, and 496 collaborations with Canada, to name the most collaborating nations. There were 412 collaborations between the United Kingdom and Italy and 279 collaborations between Italy and Spain.

### Factors Influencing CoV Research in 2020

#### Epidemiological Factors

**Figure 6:**
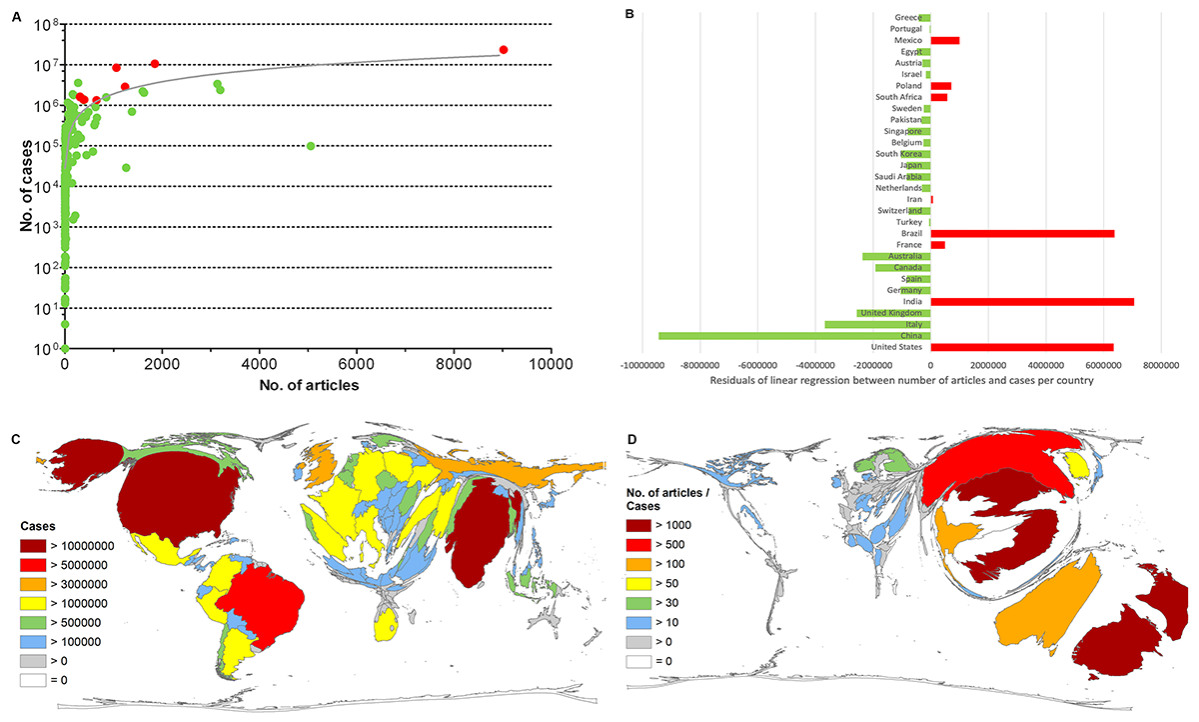
Relationship between the number of articles and COVID-19 cases per country, based on (A) linear regression (logarithmic display of y-axis), with red indicating the countries with an unfavorable ratio in terms of the number of articles among the top 30 countries; (B) residuals of the linear regression of the 30 most publishing countries, with red indicating the countries with an unfavorable ratio in terms of the number of articles; (C) density-equalizing map projection of the number of COVID-19 cases as of January 18, 2021; (D) density-equalizing map projection of the ratio between the number of articles and the number of COVID-19 cases (threshold >30 articles).

To consider the need for research on CoV according to the national burden of disease, the relationship of the number of articles on CoV to nationally registered cases of COVID-19 (as of January 18, 2021) was analyzed. The coefficient of determination was r^2^=0.64 (Figure 6A) showing a significant correlation (Spearman; *P*<.001). China showed the highest distance from the regression line (residual) toward a favorable publication performance (negative values), while India, the United States, and Brazil showed the highest deviations toward a more negative relationship (positive values). The most publishing European countries were also in the positive range of deviation from the regression line but on a lower level than China (Figure 6B).

To provide a picture of the occurrence of COVID-19 cases, a DEMP was generated showing the corresponding distortions on the world map (Figure 6C), with the highest numbers occurring in the United States, India, Brazil, Russia, the United Kingdom, France, Italy, Spain, and Germany, to indicate the countries with more than 2 million cases by January 18, 2021 (date of data collection) [3]. Relating these numbers to COVID-19 cases by calculating the ratio of countries (R_CASES_) with at least 30 articles on CoV (threshold), southeastern countries were ahead. New Zealand, with only 1900 registered cases, could be ranked first (R_CASES_=1105.26), followed by Vietnam (R_CASES_=1099.54), China (R_CASES_=512.34), Australia (R_CASES_=438.50), and Thailand (R_CASES_=119.46). On the other hand, among the most publishing countries besides China, Italy ranked next at 21st, followed by the United Kingdom (26th), Germany (30th), the United States (51st), and India (69th) due to their enormous incidence rates (Figure 6D).

#### Socioeconomic Factors

The inclusion of socioeconomic features of the publishing countries revealed a different ranking. The first ratio related the publication output per country with its economic power (ranked GDP [R_GDP_]). When looking at the countries with more than 100 articles on CoV in 2020, Jordan, as an upper-middle-income economy, led the ranking (R_GDP_=1543.10). It was followed only by high-income economies until position 21 (upper-middle-income economy=South Africa). Rank 2 could be attributed to Italy (R_GDP_=1438.54), followed by Switzerland (R_GDP_=1325.11), New Zealand (R_GDP_=1201.37), and Israel (R_GDP_=1178.45). In addition, the number of articles was significantly correlated with gross expenditures on research and development in purchasing power parity in international $ (ppp$; Spearman, *P*<.001).

In terms of the population size (R_POP_) of the same countries, only high-income countries were leading. Switzerland ranked first (R_POP_=80.08), followed by Norway (R_POP_=45.20), Israel (R_POP_=42.82), Belgium (R_POP_=42.25), and Austria (R_POP_=39.95; Table 2).

**Table 2:** Socioeconomic parameters, by country, in descending order by R_GDP_ [23].

Country	n	GDP^a^ in US $1000 billion	R_GDP_^b^	Rank R_GDP_	Population in millions	R_POP_^c^	Rank R_POP_
Jordan	133	0.09	1543.10	UMI^d^ 1	8.19	16.25	UMI 1
Italy	3195	2.22	1438.54	HI^e^ 1	62.01	51.53	HI 5
Switzerland	655	0.49	1325.11	HI 2	8.18	80.08	HI 1
New Zealand	210	0.17	1201.37	HI 3	4.47	46.93	HI 8
Israel	350	0.30	1178.45	HI 4	8.17	42.82	HI 11
United Kingdom	3135	2.79	1124.46	HI 5	64.43	48.66	HI 6
Australia	1258	1.19	1058.03	HI 6	22.99	54.71	HI 3
Denmark	270	0.26	1019.64	HI 7	5.59	48.27	HI 7
Greece	286	0.29	984.51	HI 8	10.77	26.55	HI 21
Portugal	289	0.30	972.74	HI 9	10.83	26.68	HI 20
Belgium	482	0.51	947.70	HI10	11.41	42.25	HI 12
Spain	1600	1.69	946.75	HI 11	48.56	32.95	HI 17
Singapore	441	0.49	905.73	HI 12	5.78	76.27	HI 2
Austria	348	0.42	836.74	HI 13	8.71	39.95	HI 13
Canada	1377	1.67	822.58	HI 14	35.36	38.94	HI 15
Ireland	256	0.32	789.39	HI 15	4.95	51.69	HI 4
Sweden	392	0.50	786.99	HI 16	9.88	39.67	HI 14
Netherlands	629	0.87	726.41	HI 17	17.02	36.96	HI 16
Norway	238	0.36	652.59	HI 18	5.27	45.20	HI 10
Finland	153	0.24	639.63	HI 19	5.50	27.83	HI 19
South Africa	384	0.74	521.53	UMI 2	54.30	7.07	UMI 5
Turkey	848	1.67	507.78	UMI 3	80.27	10.56	UMI 2
Hungary	135	0.27	504.48	HI 20	9.87	13.67	HI 26
United States	9018	18.56	485.88	HI 21	324.00	27.83	HI18
France	1236	2.74	451.59	HI 22	66.84	18.49	HI 25
Iran	649	1.46	444.83	UMI 4	82.80	7.84	UMI 3
Pakistan	430	0.99	435.13	LMI^f^ 1	202.00	2.13	LMI 3
Chile	181	0.44	415.04	HI 23	17.65	10.25	HI 29
Germany	1623	3.98	407.89	HI 24	80.72	20.11	HI 24
Morocco	109	0.28	385.43	LMI 2	33.66	3.24	LMI2
Romania	160	0.44	362.81	HI 25	21.60	7.41	HI 32
Saudi Arabia	623	1.73	359.91	HI 26	28.16	22.12	HI 23
Poland	368	1.05	349.81	HI 27	38.52	9.55	HI 31
Brazil	1058	3.14	337.48	UMI 5	205.82	5.14	UMI 6
Qatar	105	0.33	313.90	HI 28	2.26	46.50	HI 9
Czech Republic	109	0.35	310.63	HI 29	10.64	10.24	HI 30
Bangladesh	190	0.63	302.36	LMI 3	156.19	1.22	LMI 6
South Korea	574	1.93	297.56	HI 30	50.92	11.27	HI 28
Egypt	325	1.11	294.12	LMI 4	94.67	3.43	LMI 1
Vietnam	169	0.59	284.08	LMI 5	95.26	1.77	LMI 4
Taiwan	315	1.13	280.00	HI 31	23.46	13.42	HI 27
Malaysia	234	0.86	270.90	UMI 6	30.95	7.56	UMI 4
China	5053	21.27	237.56	UMI 7	1373.54	3.68	UMI 8
Colombia	159	0.69	230.30	UMI 8	47.22	3.37	UMI 9
United Arab Emirates	153	0.67	229.32	HI 32	5.93	25.81	HI 22
India	1847	8.72	211.79	LMI 6	1266.88	1.46	LMI 5
Argentina	173	0.88	196.73	UMI 9	43.89	3.94	UMI 7
Nigeria	211	1.09	193.76	LMI 7	186.05	1.13	LMI 7
Mexico	310	2.31	134.37	UMI 10	123.17	2.52	UMI 10
Thailand	144	1.16	124.03	UMI 11	68.20	2.11	UMI 11
Japan	605	4.93	122.67	HI 33	126.70	4.77	HI 33
Russia	269	3.75	71.83	UMI 12	142.36	1.89	UMI 12
Indonesia	187	3.03	61.76	UMI 13	258.32	0.72	UMI 13

^a^GDP: gross domestic product.

^b^R_GDP_: ratio of number of articles and GDP in US $1000 billion.

^c^R_POP_: ratio of number of articles and population in millions.

^d^UMI: upper-middle-income [23].

^e^HI: high income [23].

^f^LMI: lower-middle-income [23].

#### Funding Factors

In total, 17,590 articles received 27,150 grants from many governments and other funding agencies, including universities, hospitals, research institutions, nonprofit organizations, and private companies, among others (Table 3).

**Table 3:** Coronavirus research funding in 2020.

Funder	Number of grants
Governments	17,334
Universities/collages	4264
Trust/foundations	2004
International	967
Companies	947
Hospitals/health care	833
Societies/associations	379
Research institutes	186
Nonprofit organizations	95
Charities	52
Banks (private, nonprivate)	42
Networks/platforms	36
Parishes/churches	6
Publishers/journals	3
Unions	2

**Figure 7:**
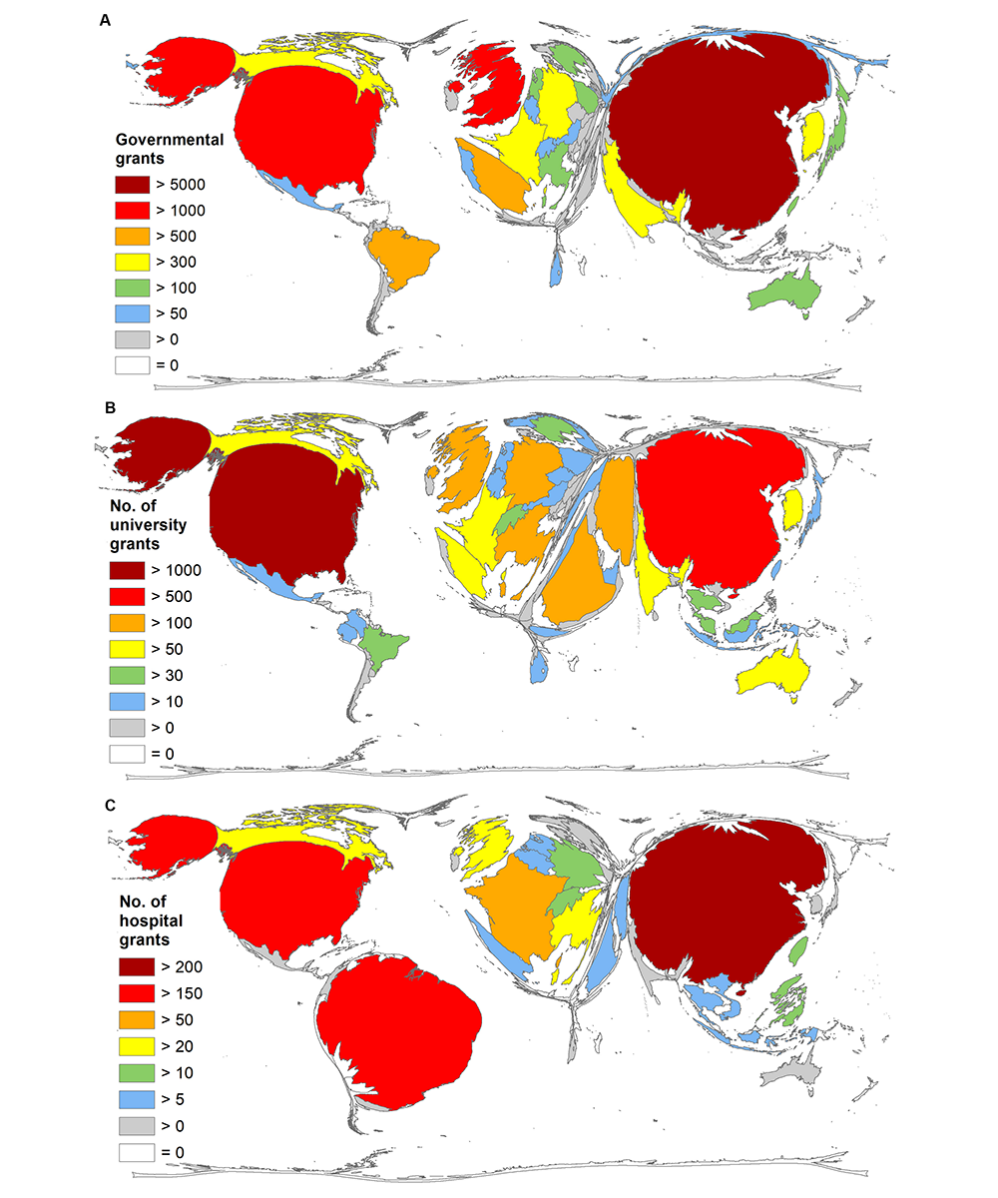
Density-equalizing maps of funding for coronavirus-related research, including (A) governmental funds, (B) university funds, and (C) hospital funds.

The government that funded the most CoV research was that of China (number of grants=6342), followed by the United States (number of grants=3983), the United Kingdom (number of grants=1179), Spain (number of grants=589), and Brazil (number of grants=504; Figure 7A). Thus, China — as the only country — had awarded more grants than published articles, resulting in a rate of 1.26 grants per article. Applying the methodological 30-article threshold, the next highest rate was achieved by South Korea (0.58 grants per article), followed by Brazil (0.48 grants per article), the United States (0.44 grants per article), and the Czech Republic (0.44 grants per article).

Looking at university funding, the United States supported the most CoV research (number of grants=1023; Figure 7B), followed by China (number of grants=899), the United Kingdom (number of grants=215), Saudi Arabia (number of grants=173), and Italy (number of grants=164). Analysis of hospital grants showed China at the top (number of grants=222; Figure 7C), followed by Brazil (number of grants=161), the United States (number of grants=160), France (number of grants=53), and Italy (number of grants=27).

In the analysis of individual funders, the US National Institutes of Health led the way, followed by the Chinese National Natural Science Foundation, the European Union, the British National Institutes for Health Research, and the Wellcome Trust based in London, United Kingdom. The top 10 funding organizations are listed in Table 4.

**Table 4:** Top 10 funders supporting coronavirus research.

Funder	Number of grants
US National Institutes of Health (NIH)	2505
National Natural Science Foundation of China (NSFC)	1467
European Union (EU)	861
UK National Institute for Health Research (NIHR)	390
French National Research Agency (ANR)	343
Wellcome Trust United Kingdom	192
Brazilian National Council for Scientific and Technological Development (CNPq)	169
Chinese Academy of Sciences (CAS)	132
Bill & Melinda Gates Foundation	120
Chinese Academy of Medical Science (CAMS)	110

## Discussion

### Principal Findings

Undoubtedly, it is not surprising that the year 2020 was marked by an immense increase in the number of publications on CoV that addressed SARS-CoV-2 infections. Nevertheless, the scale of increase is surprising: from 289 articles in 2019 to 32,402 articles in 2020 — more than a 100-fold growth.

The global COVID-19 pandemic threatens all countries’ societies with high incidence and mortality rates, so the scientific community vehemently sought solutions to contain this viral infection and the resulting symptoms through various approaches. These followed the interests of the national governments, which funded scientific endeavors just as highly.

Nevertheless, the proportion of original articles was relatively low compared to publications on other biomedical topics that are currently the talk of the town, such as climate change, where the share of articles is almost 70%, while the proportion of original research documented in CoV publications is less than 50% with a large proportion of letters and editorial material. A comparison between US-American and Chinese publications showed that the United States followed this result with nearly 70% of the article share, while Chinese articles accounted for over 90% [24]. Under the conditions of the present study, this pattern seems to be overridden in favor of shorter document types — also in the sense of rapid publication.

A closer look at the published document types also revealed that 15 papers had to be retracted although they had not even been published for a year. This demonstrates the immediacy with which results had to be published and the susceptibility to error that this occasionally entails. The pressure to be the first to publish on a new approach and deliver new results, and not be overtaken by colleagues working on the same topic, became very exigent in research on CoV in the 2020s.

The citation speed is similarly rapid. Three high-impact articles with more than 5000 citations were published in 2020 — remarkable because these papers have not yet had a year to be read or cited. The Chinese author groups reported clinical features of COVID-19 patients hospitalized in Wuhan [13-15]. Certainly, these findings have been taken up as background information for many subsequent articles.

Nevertheless, this extremely rapid publication activity, albeit at a much lower level, could also be demonstrated for the SARS and MERS epidemics, with a notable loss of research effort once the threat had subsided [4]. Had this not occurred, the state of knowledge at the onset of the SARS-CoV-2 outbreak would certainly have been sounder and the resulting measures better scientifically validated. These patterns should therefore be considered when the current pandemic has hopefully soon subsided.

It is also not surprising that in early 2020, most articles were submitted by Chinese authors reporting clinical manifestations that occurred during the outbreak in Wuhan, which was also noted in a study using the COVID-19 Open Research Dataset [25]. Subsequently, US-American researchers overtook the Chinese in terms of volume and citations received. Nevertheless, China has received the most citations so far, which is because of early publication. Articles published later have not had the time to receive so many citations. How these numbers will continue to evolve is for later studies to show. It is also not surprising that the share of Italian articles decreased slightly, considering the extreme incidence rates in Italy at the beginning of 2020. As the numbers of COVID-19 cases and mortality rates dropped, so did study numbers in Italy. As a result, Italy's share was overtaken by the British, which is not surprising due to the usually high share of British articles corresponding to the highly developed scientific infrastructure. However, the evaluation of the relationship between research performance and the number of cases showed that Italy was still in second place in the ranking of our study. Here, China was able to present itself best. The United States, which ranks first in absolute numbers in our analysis, together with India and Brazil showed a rather negative deviation due to by far the highest number of cases.

In this context, the success of the Chinese COVID-19 control must be pointed out. Certainly, the centralized epidemic response system functioning and radical surveillance had played its part. In addition, the SARS epidemic, which was accompanied by a huge mortality rate in China in 2013, was not long ago, so the awareness and compliance of the Chinese population were still very high. Both lead to an extremely fast and strict response [26,27]. In particular, in contrast to the policy response of the government of the United States, which was sparse and delayed under the regime of former President Trump, who even refused to wear a mask for a long time, China had a very quick response and was stringent with its containment measures.

Nevertheless, the US research output passed China’s during 2020. It is partly assumed that the key position of US-American science will soon be overtaken by Chinese research. However, the scientific infrastructure of the United States is profound and well prepared for rapid adaptation and ramp-up. Currently, both countries are certainly competing for the top spot in global research performance.

The dynamic nature of global CoV publication output led to the similarly dynamic development of its perception in the scientific community, resulting in enormous citation numbers already in the first year. The outbreak location in China and the reporting of hospitalized patients there with the first COVID-19 symptoms explain the high citation rate of Chinese articles, which was not presentable for other research topics.

The recognition that the virus forms a sister clade to SARS-CoV led to the taxonomy of SARS-CoV-2 by the Coronaviridae Study Group (CSG) of the International Committee on Taxonomy of Viruses. The involvement of the Netherlands in the CSG contributes to its high citation rate (rank 2 behind China) because the term is internationally accepted, and subsequent publications naturally are in unison on the designation of SARS-CoV-2 [28]. In addition to the Netherlands, Russia, among others, was also involved in this taxonomy procedure, ranking therewith third in national citation rates. Moreover, Russia, together with Germany (rank 16) and Austria (rank 4), was also involved in the highly cited cell biology study by Hoffman et al [21]. This study identifies angiotensin-converting enzyme 2 as the entry receptor and a cellular serine protease as priming the spike protein with the possibility of its blocking by proven inhibitors. Since the research field “biology and biochemistry” was identified as one of the 4 focus clusters, the importance and the level of its perception are explainable. The other key clusters addressing mental and physical health impairments as well as public health and epidemiology issues are related to the other high-impact studies identified.

When the publication numbers are related to the number of COVID-19 cases, other countries showed up in leading positions. With New Zealand, Vietnam, China, Australia, and Thailand at the top, parts of the eastern and southeastern world showed leading performances due to their very low case numbers in combination with relatively high publication numbers. Countries’ awareness of the problem influences not only the containment of the pandemic but also their research efforts and the amount of government spending on research and development. The success of achieving low case numbers was explained by the rapid implementation of nonpharmaceutical measures, determined responses, rigor and brevity of containment measures, and testing strategies by public health authorities [29,30]. Vietnam's effective response is also explained by its early preparation and strict control measures such as contact tracing, isolation, and mass testing combined with border closures. The same is true for other countries that have been successful in reducing the COVID-19 spread [31]. The success of Thailand was furthermore explained by hospitalizing any person with SARS-CoV-2 infection even without symptoms and also by demographic and environmental reasons, such as the high proportion of people living in rural areas and spending much time outdoors [32].

Africa has the lowest confirmed case rate of any continent. Only South Africa reported numbers among the 20 most-affected countries. [3] This is also reflected in the corresponding publication performance and also the related distorted maps. Beneficial demographic and geographic factors have been shown to have a significant negative correlation with the number of COVID-19 cases (eg, population density, temperature) [33].

Nevertheless, the low incidence rates in countries with poor health care systems and the associated low quality of the tests are often questioned. High risk is associated with misdeclaration or misreporting of the COVID-19 development. Therefore, globally transparent and traceable reporting is of immense importance, and the development in these regions must be closely monitored in the future [34].

On the other hand, regions with extremely high case numbers, such as the United States, India, Brazil, and some European countries, were proportionally more involved in CoV research. That is also shown by the correlation between COVID-19 cases and article numbers, which is highly significant. Only Russia, with the fourth highest case numbers, fell slightly behind but still shows an enhanced contribution rate to CoV research compared to other scientific topics.

This is also made possible by the intensive funding of the governments of the severely affected countries. In line with these figures, the high Italian case numbers prompted unusually high levels of cooperation with the United States, the United Kingdom, and Spain.

Saudi Arabia, which ranks in the middle of COVID-19 case numbers, was funded primarily through its universities, resulting in a relatively high ranking in publication numbers. This is likely due to the experience gained during the MERS epidemic, which mainly affected Saudi Arabia, accounting for 77% of cases globally. The results of an earlier analysis looking at CoV research up to the COVID-19 outbreak showed that Saudi Arabia even ranked 11th in overall publication performance [4]. Jordan, where MERS cases are also occurring, also had a prominent position in this analysis when socioeconomic characteristics were included. Other countries still affected by MERS, such as Egypt and Iran [35], are also in the field of CoV research in 2020 and are in the top 30.

### Conclusions

The awareness and preparedness of countries affected by previous CoV epidemics also led to high interest in CoV research during the 2020 COVID-19 crisis. Although disease containment may have led to a rapid decline in publication numbers, this experience appears to have been so fresh that the propensity for ad hoc research was high. On the other hand, maintaining a reasonable level of interest would have resulted in a better scientific baseline for all containment efforts at the onset of COVID-19 worldwide. This lesson learned should be kept in mind for all future research planning.

The results of this study demonstrate the extraordinary momentum of CoV research in 2020 due to the ongoing global spread of COVID-19. They also reveal the need for continued interest and dedication by scientists even after pandemics are contained. It is to expect that the next pandemic will come and also become a threat anywhere in the world. Well-prepared and sound scientific support enables decisive measures. The experience of highly developed scientific nations must be linked with that of less developed research structures to be of global benefit.

## Abbreviations

CoV: coronavirus
CSG: Coronaviridae Study Group
DEMP: density-equalizing map projections
GDP: gross domestic product
MERS: Middle East Respiratory Syndrome
NewQIS: New Quality and Quantity in Science
ppp $: purchasing power parity in current international $
R_GDP_: ranked gross domestic product
SARS: Severe Acute Respiratory Syndrome
WoS: Web of Science
